# In-office laryngeal procedures (IOLP) in Canada: current safety practices and procedural care

**DOI:** 10.1186/s40463-018-0270-2

**Published:** 2018-04-03

**Authors:** Yael Bensoussan, Jennifer Anderson

**Affiliations:** 1 Department of Otolaryngology Head & Neck Surgery, University of Toronto, Toronto, ON Canada; 2 Department of Otolaryngology-Head and Neck Surgery, University of Toronto, St-Michael’s Hospital, 30 Bond Street, Toronto, ON M5B 1W8 Canada

**Keywords:** Office-based procedures, Patient safety, Laryngology procedures, Awake procedures, Patient tolerance, Complications

## Abstract

**Background:**

The advent of chip tip technology combined with advanced endoscopy has revolutionized the field of laryngology in the past decade. Procedures such as transnasal esophagoscopy, site-specific steroid injections, injection laryngoplasty and laryngeal laser treatment can now be performed in the office setting under local anaesthesia. Although In-Office Laryngeal Procedures (IOLPs) have become standard-of-care in many American and several Canadian centers, there are no guidelines regulating the practice of these procedures. The goal of this report was to evaluate the current method of IOLP delivery in Canada.

**Methods:**

An electronic survey was dispersed to 22 practicing Canadian laryngologists to assess safety and procedural care measures undertaken when performing IOLP. The survey consisted of 37 questions divided into 6 categories; 1) Demographic data 2) Facilities 3) Staff/personnel 4) Patient screening/monitoring 5) Procedure and emergency equipment 6) Reporting of adverse events.

**Results:**

Data was collected for 16/22 laryngologists (72.7% response rate). Only 1 respondent did not perform IOLP. All performed injection augmentation laryngoplasty. Most performed laryngeal biopsies, intramuscular injection and/or electromyography guided injection for the treatment of spasmodic dysphonia and glottic/subglottic steroid injections. Only 4 respondents performed in-office KTP laser. Significant variation was found in procedural processes including intra procedural monitoring, anticoagulation screening, access to emergency equipment and documentation.

**Conclusion:**

Our survey demonstrates that the delivery of IOLP in Canada varies considerably. The construct of IOLP practice guidelines based on the evidence with consistent documentation would promote safe, efficient and quality care for patient with voice disorders.

**Electronic supplementary material:**

The online version of this article (10.1186/s40463-018-0270-2) contains supplementary material, which is available to authorized users.

## Background

The advent of chip tip technology combined with advanced endoscopy including port access has revolutionized the field of laryngology in the past decade [1–4]. Procedures such as laryngeal biopsies, transnasal esophagoscopy, steroid injection, injection augmentation and laryngeal laser treatment can be performed in the office setting on awake patients with videoendoscopic guidance.

In office laryngology procedures (IOLP) have specific advantages over traditional surgical management for the treatment of laryngeal pathology such as improved access, shorter procedure time and less cost [4–6]. In safety measures, IOLP with local/topical anaesthesia also represent reduced risk by avoiding general anaesthesia and other surgery associated risk inherent to suspension laryngoscopy (injury to dentition, tongue/mucosal, TMJ/jaw). In patients with significant comorbidities or anatomic limitations who are not suitable or are at a high risk of complications to undergo a general anaesthetic with suspension laryngoscopy, IOLP is a viable alternative to treatment which was not available previously.

Disease outcomes, safety, and tolerance of these procedures in awake patients have been explored in the literature [4–7]. Although IOLP have now become ‘routine’ practice in many American and several Canadian centers, to date, there are no guidelines regulating the practice of these procedures despite potential adverse events and complications.

The purpose of this study was to report the current practices in terms of safety measures and procedural processes for IOLP in the Canadian health system.

## Methods

An electronic survey was dispersed to 22 practicing Canadian laryngologists trough an electronic survey platform to assess safety and procedural measures undertaken when performing IOLP. Canadian laryngologists were identified through the membership list of the Canadian Society of Otolaryngology – Head & Neck Surgery (CSOHNS) (self-reported laryngologists) and by contacting Otolaryngology departments of each Canadian university. The list was then reviewed by the senior academic laryngologist (J.A) at our institution and recent graduates were added to the list. Once the list was completed, laryngologists were contacted by email and/or phone and received a link to complete the anonymous survey through the electronic platform. Data was collected in a cross-sectional fashion between November 2016 and June 2017.

The survey consisted of 37 questions divided into 6 categories; 1) demographic data 2) facilities 3) staff/personnel 4) patient screening/monitoring 5) procedure and emergency equipment 6) monitoring and reporting. After the demographic section, participants were asked if they performed IOLP. A negative answer ended the questionnaire, whereas a positive answer opened further questions. The full survey questions are available in the electronic version of this manuscript in Additional file 1.

## Results

The survey was completed by 16 of the 22 laryngologists (72.7% response rate) with an equal number or male and female laryngologists. The majority of the respondents were fellowship trained (87.5%). Complete demographics data is summarized in Table 1.

**Table 1:** Demographic data of participating Canadian Laryngologists

Variable	Respondents *N* = 16 (%)
Gender	
Male	8 (50.0)
Female	8 (50.0)
Province of practice	
Ontario	7 (43.8)
Alberta	3 (18.8)
Manitoba	2 (12.5)
British Columbia	2 (12.5)
Quebec	1 (6.25)
Nova Scotia	1 (6.25)
Years in practice	
Less than 5 years	5 (31.3)
5 to 10 years	4 (25.0)
10 to 20 years	3 (18.8)
More than 20 years	4 (25.0)
Fellowship training	
Yes	14 (87.5)
No	2 (12.5)

Fifteen out of 16 respondents (93.8%) reported performing IOLP. Only 1 respondent did not perform IOLP despite fellowship training due to concerns raised from their hospital administration about safety for these procedures. As such, the remaining questions of the survey were answered by 15 respondents.

**Fig. 1:**
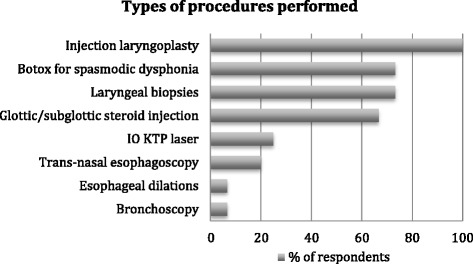
Procedures. Types of procedures performed by Canadian Laryngologists respondents

Injection laryngoplasty was the most performed procedure (15/15), followed by laryngeal biopsies (11/15), botulinum toxin A injections for spasmodic dysphonia (11/15) and video endoscopic guided glottic/subglottic steroid injection (10/15). Four respondents reported performing in office KTP laser in their clinic which were all hospital-based. Other procedures included transnasal esophagoscopy, esophageal dilation, and bronchoscopy as summarized in Fig. 1.

### Facilities

All respondents reported performing IOLP in a clinic within a hospital. One third (33.3%) of laryngologists also perform the procedures out-of hospital in a private clinic or office outside the main hospital facility, between 2 to 4 km away from their base hospital.

### Staff/personnel

Fifty three percent (8/15) respondents indicated that their assistant/nursing staff had received some form of IOLP related training. Human resources available for assistance to perform IOLP was variable and included registered nurses, otolaryngology trainees, speech language pathologist and physician assistants. On the other hand, 80.0% of respondents indicated their staff/assistants have training for emergency situations such as CPR (cardiopulmonary resuscitation) or ACLS (advanced cardiovascular life support) training.

### Patient screening and monitoring

Participants were asked to describe if there was exclusion criteria used to screen patients before IOLP (Table 2.). Three laryngologists (20.0%) indicated there had no exclusion criteria for these types of procedures.

**Table 2:** Exclusion criteria reported

Exclusion criteria	Number of times reported (*n* = 15)
Intolerance to office scope/ severe gag	6
Significant anxiety	4
Poor Anatomy/obesity	4
Poor lung function/02 requirement	2
Lesion too bulky for KTP	2
Neuromuscular disease	1
Uncontrolled hypertension	1
Allergy to lidocaïne	1
Unable to understand English	1
None	3

The survey results demonstrated considerable variability in whether or not patients were instructed to discontinue anticoagulation medication by their laryngologist prior to undergoing IOLP (Table 3). More than 20% of respondents reported performing IOLP for patients on therapeutic levels of warfarin. However, none of these laryngologists were performing laser treatment.

**Table 3:** Antiplatelet/anticoagulation management

	Would Continue *N* = 15 (%)	Would Stop^a^*N* = 15 (%)	Would perform IOLP if patient cannot stop
ASA 81 mg	11 (73.3)	4 (26.7)	15 (100.0)
NSAIDs	12 (80.0)	3 (20.0)	13 (86.7)
Clopidogrel	5 (33.2)	10 (66.7)	7 (46.7)
New agents (i.e. dabigatran)	8 (53.5)	7 (46.7)	5 (33.3)
Warfarin	3 (20.0)^b^	12 (80.0) ^c^	4 (26.7)^d^

^a^ Stop from 3 to 7 days prior to procedure

^b^ 1 respondent specified if INR less than 3.5

^c^ With or without bridging

^d^ 1; only for injection laryngosplasty, 1; only if INR over 2.5 (hold or modify)

Sixty per cent (9/15) of laryngologists use pre-medication such as an anxiolytic (i.e. lorazepam) for some patients whereas 40.0% indicated that premedication or sedation was never used.

The majority of the laryngologists (86.7%) did not require patients to be NPO before their procedure.

Only 35.7% of laryngologists reported systematically measuring and documenting vital signs as part of their procedure protocol. In terms of patient monitoring, only 1 laryngologist reported taking post-procedure vital signs.

Most centres monitored patients post IOLP for 15–30 min (73.3%), and in some cases, less than 15 min (20.0%) and rarely over 30 min (6.7%).

Most laryngologists always ask patients to be accompanied after a procedure (66.7%). Two of the three laryngologists who reported not requiring that patients be accompanied perform full range of procedures described previously including KTP laser procedures (Table 4).

**Table 4:** Procedural care questions

Procedural care	Respondents *n* = 15 (%)
Do you use sedation/pre-medication?	
Yes, for anxious patients	9 (60.0)
No, Never	6 (40.0)
Do you ask your patient to be NPO before the procedure?	
Yes	2 (13.3)
No	13 (86.7)
How long are patients monitored post-procedure?	
Less than 15 min	3 (20.0)
15–30 min	11 (73.3)
More than 30 min	1 (6.7)
Do you ask patients to be accompanied post procedure?	
Yes	10 (66.7)
No	3 (20.0)
Depending on procedure	1 (6.7)
Only if pre-medicated	1 (6.7)

### Procedure and emergency equipment

The majority of laryngologists surveyed use chip tip video endoscopy (93.3%), and 40% also use fiberoptic scopes to perform IOLP. A variety of local anaesthesia techniques were reported. For nasal anaesthesia, half of the respondents use packing whereas the remaining use spray technique. The agents used include lidocaine, or a mix of lidocaine and xylometazoline. For laryngeal anaesthesia, transcutaneous/transtracheal injection of lidocaine and topical lidocaine delivered via a port catheter in the endoscopy were the most commonly used techniques (60.0% and 53.3%). Other methods less often utilized were the use of nebulizer/mist inhaled lidocaine, endotracheal lidocaine spray, laryngojet and superior laryngeal nerve block.

**Fig. 2:**
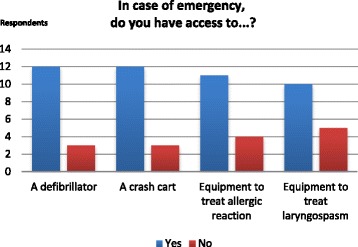
Availability of Emergency Equipment

In terms of emergency equipment, 80.0% of respondents have access to a crash cart and defibrillator, 73.3% have access to material to treat an allergic reaction, and 66.7% have access to material to treat a laryngospasm in the case of severe complication (Fig. 2). Out of the 3 respondents who said not having a crash cart in their facility, 1 reported he had access to one 500 m away from the clinic facility.

### Monitoring and reporting of adverse events

In an open-ended question about adverse events and complications encountered by laryngologists, only minor complications were reported such as patient anxiety, intolerance, intractable gag, coughing, vomiting, vasovagal episodes, discomfort and pain. One laryngologist reported a minor laryngospasm adverse event, spontaneously resolving after termination of the procedure and patient monitoring.

Eleven out of 15 physicians (73.3%) document of the dosage of topical and/or injected local anaesthesia used during the procedure. All surveyed laryngologists dictate and/or write procedure notes in the medical chart. In addition, two respondents also have a nursing note added to the chart. One laryngologist keeps a separate binder for injectables where dosages and materials are documented. One respondent used a standardized laser flow sheet and safety checklist for all laser procedures.

In terms of reporting of adverse events, all respondents indicated that the patients’ electronic or paper medical record was used for documentation. One respondent also uses a separate adverse event hospital log for standardized reporting.

## Discussion

The purpose of this national survey was to describe the procedural practices used to conduct IOLP in Canada. This initiative was motivated by the fact that there are presently no guidelines on procedure and safety measures for IOLP.

Operating room procedures are regulated by detailed protocols including surgical timeout and debriefing, instruments counts, nursing and anaesthesia monitoring, and laser safety protocol as well as standardized documentation [8–11]. Although patients are not undergoing general anaesthesia, in-office laryngology procedures have potential significant risks.

Any use of sedation or local anaesthesia poses a potential risk of allergic reaction or toxicity [12]. Moreover, the specific risks of airway manipulation of the awake, non-intubated patients are particularly of concern in our specialty. Although largely minor complications such as vasovagal episodes, minor airway bleeding, discomfort, pain, patient intolerance are reported in the literature, some authors have encountered laryngospasm, airway bleeding, and lidocaine toxicity during these procedures [2, 3, 6, 7, 13]. More recent reports from several authors describe non-negligible hemodynamic effects during IOLP including severe tachycardia and hypertension [14–16].

Our survey results suggest that although most laryngology practices are equipped in case of adverse events, a significant percentage of respondents (20.0–33.3%) reported no access to medical resources in order to treat laryngospasm or allergic reactions. In case of an emergency situation when in a hospital setting, any serious adverse event can be supported through usual emergency procedure I.E. code blue. However, immediately accessible emergency resources such suction (bleeding, secretions), arrest cart (standardized medical treatment) and defibrillator are reasonable for patient safety.

In an office setting outside the hospital, access to these resources may be limited or unavailable. One third of the responding laryngologists reported practicing in such a setting.

When looking at the literature from other specialties performing office- based procedure, several associations of surgeons have published guidelines to regulate these practices and ensure patient safety. The American Academy of Dermatology recently published explicit guidelines for the use of local anaesthesia in office-based dermatological surgery (2016) [17]. These guidelines detail evidence on types of agents, methods of delivery, and potential complication. Among other recommendations, they suggest calculation and documentation of local anaesthetic use for each patient to prevent adverse toxicity events. The Society of American Gastrointestinal Endoscopic Surgeons (SAGES) has also issued guidelines regulating endoscopy practices [18]. The published document includes recommendations in terms of facilities and physical environment, training of staff, patient selection and NPO status, patient monitoring, equipment, medication requirements and documentation.

Although there are no published guidelines in laryngology, several authors have recommended some measures to promote patient safety. Madden et al. suggested a cardiovascular pre-screening tool to prevent avoidable complications for high risk patients [19]. The protocol consists of measuring vital signs at the pre-operative appointment to screen patients and refer to the appropriate physician before exposing patients to avoidable risks. Yung et al. also suggested the monitoring of vitals during these procedures after reporting significant changes in BP and HR [14]. The results of the present survey suggests that less than 40% of Canadian laryngologists measure vital signs prior to or after IOLP. There is no current data that describes American laryngologists practices in terms of vital signs monitoring during these procedures.

The analysis of the survey responses also demonstrates that similar to other surgical specialties, office based procedures are increasingly being performed in laryngology in Canada. Brown et al., from Halifax, had already described the trend in 2012 by reporting the results of a survey detailing the composition of practice and technology used by Canadian laryngologists [20]. At that time, the authors identified 11 laryngologists in Canada. According to our data, the self-reported laryngologists in practice have doubled in the last 5 years (*n* = 22) with one third of out respondents in practice less than 5 years. The variety and complexity of procedures performed on awake patient have also increased with now at least 4 laryngologists performing in-office KTP laser in Canada.

There may be some influence on delivery of novel procedures due to resource restrictions and physician remuneration based on provincial fee schedules. For example, in eastern Canada and a few academic centres in Ontario, physicians are on a full salary and are not limited by the lack of a specific procedure code in offering these novel treatment options. However, in western Canada (British Columbia and Alberta) there is no specific fee code for in office laser treatment. Laryngeal augmentation and botox injection however have has a code in the schedule in most provinces. In Ontario and Quebec, a specific business case was submitted to the provincial health organization which approved in office laser laryngeal procedures. It is important to take into consideration safety requirements may vary between institutions as well. The survey did not have specific questions regarding differences in institutional restrictions or provincial remuneration.

Another limitation of this study is the relative risks of the various types of IOLP. For example. A 30 min endoscopic laser treatment for extensive RRP likely does not have the same degree of risk as a unilateral injection for spasmodic dysphonia (usually less than 5 min) which may therefore influence exclusion criteria or the need for cessation of anticoagulants prior to procedures. However, all the IOLP listed in our survey require manipulation of the upper airway in an awake patient and safety measure such as access to emergency equipment, training of assistants, and standardized reporting of adverse events are recommended.

## Conclusion

IOLPs are increasingly practiced in the laryngology field in the Canada. Our survey demonstrates that practices in terms of safety and procedural care remain variable. Practice enhancement strategies should focus on providing more structured guidelines to regulate IOLP and promote patient safety.

## Additional file

Additional file 1: Current safety practices for In-Office laryngology procedures: A Canadian Survey. (DOCX 23 kb)
