# Supplementary material for: In-office laryngeal procedures (IOLP) in Canada: current safety practices and procedural care
Source: J Otolaryngol Head Neck Surg. 2018 Apr 3;47:23. doi: 10.1186/s40463-018-0270-2 (PMC5883580; doi:10.1186/s40463-018-0270-2)
Supplement: Supplementary file 1 — Current safety practices for In-Office laryngology procedures: A Canadian Survey. (DOCX 23 kb) [file 40463_2018_270_MOESM1_ESM.docx]

Current safety practices for In-Office laryngology procedures: A Canadian Survey

Dear doctor,

You are being asked to participate in this study because you are laryngologist practicing in Canada.

We are presently conducting a survey on safety practices and procedural care for in-office laryngeal procedures. As you know, these procedures have gained popularity in the last decade due to their cost-effectiveness for many laryngeal pathologies. Unfortunately, many cases of morbidity and a few cases of mortality have raised safety concerns for in-office laryngology procedures.

We are asking you to complete this survey to grasp the current safety practices and assess the need for detailed protocols to ensure patient safety during these procedures.

We thank you in advance for your help and collaboration.

**SECTION A**

1. Are you a:

- Male?
- Female?

1. Years in Practice?
   - - <5
     - 5-10
     - 10-20
     - >20
2. In which province do you practice?
3. Are you fellowship trained in Laryngology?
   1. If yes please provide fellowship program
4. Do you perform In-office laryngeal procedures in your practice?

- IF YOU ANSWERED NO – only complete sections **a + B**
- IF you answered YES – COmplete sections **A + C + D**

**SECTION B**

1. If you answered NO to question 5 – Please chose reason:

- I do not feel I have enough training to perform in-office laryngology procedures
- My facility does not allow me to perform in-office procedures
- I do not feel I have the resources to perform in-office laryngology procedures
- Other :

**SECTION C**

1. If you answered yes to question 5 - please select in-office procedures:
   - Transnasal fiberoptic laryngoscopy guided laryngeal biopsies
   - Injection laryngoplasty
   - Glottic/subglottic Steroid injections
   - Botox injections for spasmodic dysphonia
   - Videoendoscopic guided KTP laser
   - Other:

**SECTION D**

**Facilities**:

1. What facility do you perform in-office laryngeal procedures in?
   - In a clinic within a hospital
   - In a clinic outside the hospital
     - How far is the closest hospital (km)?
   - In private office

**Personnel:**

1. Does your nursing staff/clerks/assistants have specific training in in-office procedures?
   - Please specify:
2. Does your nursing staff/clerks/assistant have specific training for emergency situations (CPR/ACLS)?
   - Please specify

**Patients**:

1. Do you have selection criteria for patients for possible in-office laryngeal procedures?
2. How do you screen/instruct patients prior to their procedure on anticoagulation use? (ex: ASA? Plavix?)
3. Do you recommend patients are NPO on day of procedure?
4. Do you have any exclusion criteria? (Age, specific comorbidity, allergy, anxiety)
5. Do you have a pre-procedural protocol? i.e. vital signs Vital signs? Please describe.
6. What does the usual post-procedural protocol include?
   1. How long are patients observed post procedure?
   2. Do you ask for someone to bring them home?

**Material/equipment**

1. How do you store the drugs used for in-office procedures:

- Topical anesthetic i.e. Lidocaine:
- Botox:
- Radiesse:

1. Who is responsible for the equipment cleaning and maintenance (scopes, laser, fibers?)
2. Do you have a crash cart in your facility? How far is the closest crash cart?
3. Do you have access to a defibrillator in case of cardiac arrest?
4. Do you have access to equipment in case of an allergic reaction?
   - Please specify:
5. Do you have access to equipment/material/drugs to treat laryngospasm?
   - Please specify:

**Monitoring**

1. Where do you record details of the procedure?
2. Do you keep record the dose of topical anaesthetic used?
3. How are adverse events documented?

**Complications/patient tolerance**

1. Please describe any complications, adverse events or situations where you had to abort an in-office laryngeal procedure. (ex: Laryngeal spasm, vocal fold haemorrhage, intractable gag reflex, anxiety, etc.)

**Thank you for taking the time to complete our survey and contributing to quality improvement research!**
